# Two Acidic, Anticoagulant PLA_2_ Isoenzymes Purified from the Venom of Monocled Cobra *Naja kaouthia* Exhibit Different Potency to Inhibit Thrombin and Factor Xa via Phospholipids Independent, Non-Enzymatic Mechanism

**DOI:** 10.1371/journal.pone.0101334

**Published:** 2014-08-13

**Authors:** Ashis K. Mukherjee, Bhargab Kalita, Rupamoni Thakur

**Affiliations:** Microbial Biotechnology and Protein Research Laboratory, Department of Molecular Biology and Biotechnology, School of Science, Tezpur University, Tezpur, Assam, India; Universidad de Costa Rica, Costa Rica

## Abstract

**Background:**

The monocled cobra (*Naja kaouthia*) is responsible for snakebite fatality in Indian subcontinent and in south-western China. Phospholipase A_2_ (PLA_2_; EC 3.1.1.4) is one of the toxic components of snake venom. The present study explores the mechanism and rationale(s) for the differences in anticoagulant potency of two acidic PLA_2_ isoenzymes, Nk-PLA_2_α (13463.91 Da) and Nk-PLA_2_β (13282.38 Da) purified from the venom of *N. kaouthia*.

**Principal Findings:**

By LC-MS/MS analysis, these PLA_2_s showed highest similarity (23.5% sequence coverage) with PLA_2_ III isolated from monocled cobra venom. The catalytic activity of Nk-PLA_2_β exceeds that of Nk-PLA_2_α. Heparin differentially regulated the catalytic and anticoagulant activities of these Nk-PLA_2_ isoenzymes. The anticoagulant potency of Nk-PLA_2_α was comparable to commercial anticoagulants warfarin, and heparin/antithrombin-III albeit Nk-PLA_2_β demonstrated highest anticoagulant activity. The anticoagulant action of these PLA_2_s was partially contributed by a small but specific hydrolysis of plasma phospholipids. The strong anticoagulant effect of Nk-PLA_2_α and Nk-PLA_2_β was achieved via preferential, non-enzymatic inhibition of FXa (*Ki* = 43 nM) and thrombin (*Ki* = 8.3 nM), respectively. Kinetics study suggests that the Nk-PLA_2_ isoenzymes inhibit their “pharmacological target(s)” by uncompetitive mechanism without the requirement of phospholipids/Ca^2+^. The anticoagulant potency of Nk-PLA_2_β which is higher than that of Nk-PLA_2_α is corroborated by its superior catalytic activity, its higher capacity for binding to phosphatidylcholine, and its greater strength of thrombin inhibition. These PLA_2_ isoenzymes thus have evolved to affect haemostasis by different mechanisms. The Nk-PLA_2_β partially inhibited the thrombin-induced aggregation of mammalian platelets suggesting its therapeutic application in the prevention of unwanted clot formation.

**Conclusion/Significance:**

In order to develop peptide-based superior anticoagulant therapeutics, future application of Nk-PLA_2_α and Nk-PLA_2_β for the treatment and/or prevention of cardiovascular disorders are proposed.

## Introduction

The Indian monocled cobra (*Naja kaouthia*) is one of the major venomous snakes of the Elapidae family. This species of snake is responsible for major snakebite fatality in India, Bangladesh, Nepal, Myanmar, and south-western China [1]. Snakebite remains a neglected tropical disease which requires immediate medical attention. The venom of *N. kaouthia* is enriched with various enzymes and non-enzymatic protein/peptide toxins [1]. Among the snake venom enzymes, PLA_2_s, because they have a crucial role in inducing various pharmacological effects on snakebite victims, are an interesting group of proteins [2], [3]. Snake venom PLA_2_ enzymes exhibit a wide variety of pharmacological effects such as neurotoxicity, cardiotoxicity, cytotoxicity, membrane damaging activity, myotoxicity, necrotic, anticoagulant, hemolytic, haemorrhage and edema induction by various mechanisms of action [2]–[11]. The structure-function relationship and the mechanism of action of this group of small proteins are still subtle, complex and thus impose intriguing challenges to the toxinologists [2], [12].

The systemic neuromuscular blockade and other clinical signs of *N. kaouthia* envenomation have been described. However, the mechanism(s) of anticoagulant action of snake venom PLA_2_ enzymes still remains incomprehensible and a detailed molecular mechanism of anticoagulant action of PLA_2_ enzymes from *N. kaouthia* venom has never been explored. Our previous studies have shown the biochemical and pharmacological characterization as well as membrane damaging activities of major PLA_2_ isoenzymes purified from the venom of *N. kaouthia* of different geographical origins [5]–[7]. These Nk-PLA_2_s were found to prolong the Ca-clotting time of citrated platelet poor plasma [7]; however, their mechanism of anticoagulant action was not explored. Snake venom PLA_2_ enzymes may prolong the blood coagulation in victims by different ways; either by enzymatic hydrolysis of plasma phospholipids which is required to initiate the coagulation process and/or binding with essential blood coagulation factors through a non-enzymatic mechanism [2]–[3], [8]–[9]. Studies on the mechanism of such anticoagulants will advance our understanding of ‘vulnerable’ sites in the coagulation cascade [12]. This study may further help us to design novel strategies towards developing anticoagulant therapeutics for treating cardiovascular disorders (CVDs) and also new functional diagnostic test kits in the field of haemostasis [2], [10], [13].

In the present study, we report the purification and the mechanism of anticoagulant action of two major PLA_2_ enzymes purified from the venom of *N. kaouthia* of eastern India origin.

## Materials and Methods

The crude *N. kaouthia* venom sample (pooled) was obtained from authorized, licensed venom dealer Mr. D. Mitra, Calcutta Snake Park, Kolkata [5], [6], [8]. Lyophilized monovalent antivenom (against *N. kaouthia* venom) was obtained from Vins Bioproducts Limited, India (batch no: 30AS11001; expiry date: 04/2015). All other chemicals used were of analytical grade and were procured from Sigma-Aldrich, USA.

### Purification of two PLA_2_ isoenzymes

All the fractionation steps were carried out at 4°C. Twenty five mg (dried weight) of *N. kaouthia* crude venom, dissolved in 0.5 ml of 20 mM Tris-HCl, pH 7.4, was injected to a FPLC-HiPrep CM FF16/10 column (20 ml) pre-equilibrated with 20 mM Tris-HCl, pH 7.4 (buffer A). The column was coupled to a Fast Protein Liquid Chromatography (FPLC) system (Akta Purifier 10, Wipro GE Healthcare). After washing the column with 30 ml (1.5 CV) of equilibration buffer, the bound proteins were eluted from 0 to 50% linear gradient of buffer B (20 mM Tris-HCl, pH 7.4 containing 1M NaCl) for 100 min at a flow rate of 1.0 ml/min. One ml fraction was collected in each tube. The elution of protein was monitored at 280 nm and the peaks were then screened for protein content [14], PLA_2_ (see below), and plasma clotting activities (see below).

The cation-exchange unbound fractions showing maximum anticoagulant as well as PLA_2_ activities were pooled, desalted, lyophilized, dissolved in 0.5 ml of 20 mM Tris-HCl, pH 7.4 and was then applied to an anion exchange FPLC Hiprep DEAE FF16/10 column (20 ml) pre-equilibrated with the above buffer. After washing the column with 30 ml of equilibration buffer (1.5 CV), bound proteins were eluted from 0 to 50% linear gradient of buffer B (20 mM Tris-HCl, pH 7.4 containing 1M NaCl) for 100 min at a flow rate of 1.0 ml/min. One ml fraction was collected in each tube. The elution of protein was monitored at 280 nm, and the peaks were then screened for protein content [14], PLA_2_, and plasma clotting activities. The purity and molecular mass of 1.0 µg protein of pooled fractions showing high PLA_2_ and anticoagulant activities were determined by MALDI-TOF-MS (4800 Plus MDS SCIEX, Applied Biosystems) as described previously [3], [15].

### Protein identification by LC-MS/MS

Twenty five microgram of the lyophilized sample after reduction and alkylation was subjected to in-solution digestion with proteomics grade trypsin (Promega) for overnight at 37°C [15]. The digested peptides were reconstituted in 15 µl of the 0.1% (v/v) formic acid and were subjected to RP-HPLC-MS/MS analysis with collision induced dissociation as the fragmentation method. The data so generated was searched for the identity of tryptic peptides using MASCOT 2.4 search engine on a Proteome discoverer 1.4 platform. The data was searched against Uniprot Swiss-Prot database (non redundant database with reviewed proteins) and Uniprot TrEMBL (database with unreviewed proteins) database against the taxid snake from NCBI. Minimum of two high confident peptides was used as a prerequisite to identify the protein(s) of interest.

### PLA_2_ activity assay

The PLA_2_ activity was assayed as described previously [5], [8] by using egg-yolk phospholipids as substrate. One unit of PLA_2_ activity has been defined as the amount of protein which produces a decrease in 0.01 absorbance after 10 min at 740 nm. The substrate specificity of purified PLA_2_ was determined by using different commercially available phospholipids (1 mM) viz. phosphatidylcholine (PC), phosphatidylserine (PS) and phosphatidylethanolamine (PE). The enzyme activity was assayed by titrametric method with palmitic acid as a free fatty acid standard [6], [8]. The PLA_2_ activity is expressed as ng of fatty acid released/min/mg of protein.

### Anticoagulant activity assay

Platelet poor plasma (PPP) was used for anticoagulant activity assay [3], [8]. One unit of anticoagulant activity has been arbitrarily defined as one second increase in clotting of PLA_2_-treated PPP compared to the clotting time of control PPP [5], [8]. To determine the dose-dependent anticoagulant activity different amounts (0.1 to 1.0 µg) of Nk-PLA_2_ were incubated with PPP (300 µl) for 3 min at 37°C followed by an addition of 40 µl of 250 mM CaCl_2_ in order to initiate the clot formation. As a positive control, the anticoagulant activity of heparin and warfarin was also determined under identical experimental conditions. The effect of pre-incubation on anticoagulant activity was determined by pre-incubating 300 µl PPP with 0.5 µg PLA_2_ for 5–30 min at 37°C and then the anticoagulant activity was determined as stated above.

To determine the release of PLA_2_-induced free fatty acids from PPP, 0.5 µg PLA_2_ (dissolved in 20 mM Tris-HCl, pH 7.4) was pre-incubated with PPP (300 µl) for 3–30 min at 37°C, and thus the liberated fatty acids were extracted and micro titrated by using 0.01 N NaOH and phenolphthalein red as indicator [8]. A control (PPP treated with buffer) was also run in parallel.The fatty acids liberated from the PPP (treated with PLA_2_ or buffer) were extracted and quantified as described by Doley et al. [6].

### Thrombin and Factor Xa inhibition assay

To observe the effect of purified PLA_2_ isoenzymes on amidolytic activity of thrombin or FXa, purified PLA_2_ (1.0 µg in 20 mM Tris-HCl, pH 7.4) was pre-incubated with thrombin (0.03 NIH U/ml) or FXa (0.15 µM) for 30 min at 37°C and then by assaying the activity of these coagulation factors against their chromogenic substrates (0.2 mM) T1637 and F3301, respectively [3], [8]. The release of p-nitroaniline (pNA) was monitored for 15 min at intervals of 1 min at 405 nm in a plate reader (Multiskan™ GO, Thermo Scientific, USA). For every experiment, a control was run in parallel. The activity of thrombin or FXa towards their substrates was considered 100% activity and other values were compared with this.

The dose-dependent (0–0.2 µg) and time-dependent (5–30 min) antithrombin activity of purified PLA_2_ against fibrinogen (7.5 µM), which is the physiological substrate for thrombin, was also determined as described previously [3]. In another set of experiments, the FXa inhibition assay was performed by pre-incubating 20 nM FXa with 1.0 µg of PLA_2_ for 60 min at 37°C and then the reaction was initiated by adding prothrombin (1.4 µM) to this mixture. The prothrombin activation was determined by formation of thrombin using its chromogenic substrate T1637 [8]. Thrombin formation was also determined by 15% SDS-PAGE analysis of prothrombin activation products after 1 h incubation of prothrombin (10.0 µg) with 20 nM FXa (pre-treated with PLA_2_ or buffer) at 37°C, pH 7.4. The formation of thrombin was confirmed by the peptide mass fingerprinting analysis of ∼36 kDa protein. For each experiment, a control was also run in parallel where buffer instead of PLA_2_ was added.

### Determination of inhibitory constant (K_i_) for the inhibition of FXa and thrombin

For determining the inhibitory constant (*K*i value) of Nk-PLA_2_α on amidolytic activity of FXa, a fixed concentration of FXa (20 nM) was pre-incubated with two different concentrations of Nk-PLA_2_α (50.0 nM and 100.0 nM) in a 96-well microplate at 37°C for 60 min. A control was run in parallel where FXa was incubated with buffer (20 mM Tris-HCl, pH 7.4) under identical experimental conditions. Then graded concentrations (0.1–0.8 mM) of chromogenic substrate for factor Xa (F3301) were added and the final volume of reaction was adjusted to 100 µl with 20 mM Tris-HCl, pH 7.4. After incubation for 10 min, the release of pNA was determined at 405 nM. For kinetic analysis, the reaction rate (V) was plotted against substrate concentrations (S) at each inhibitor concentration and the data was fitted to a hyperbolic Michaelis-Menten model using GraphPad Prism 5.0 software. The inhibitory constant (*Ki*) was determined by using the uncompetitive model (shown below) for enzyme inhibition by using the same software.
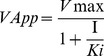
(1)

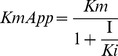
(2)

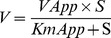
(3)


In the above equations (1) and (2), I indicates the inhibitor concentration; *VApp* and *Vmax* represent maximum velocity in the presence and absence of the inhibitor, respectively. The *KmApp* and *Km* in equation 2 denote the Michaelis constant in the presence and absence of inhibitor, respectively.

The *Ki* value for inhibition of amidolytic activity of thrombin by Nk-PLA_2_β was determined by pre-incubating a fixed concentration of thrombin (0.03 NIH U/ml) with two different concentrations of Nk-PLA_2_β (50.0 nM and 100.0 nM) at 37°C, pH 7.4 for 30 min. In control, thrombin was incubated with buffer (20 mM Tris-HCl, pH 7.4) instead of Nk-PLA_2_β under identical experimental conditions. The reaction was initiated by adding graded concentrations (0.1–0.8 mM) of chromogenic substrate for thrombin (T1637) in a final reaction volume of 100 µl adjusted with 20 mM Tris-HCl, pH 7.4. After incubation for 10 min at 37°C, the release of pNA was determined at 405 nM in a microplate reader. The kinetics analysis was done by using the GraphPad Prism 5.0 software and the *Ki* value was determined as stated above. The experiments were repeated three times to assure the reproducibility.

### Spectrofluorometric assay of interaction of PLA_2_ with thrombin/FXa and PC

To study the protein-protein interaction, thrombin/FXa was pre-incubated with NK-PLA_2_ (1∶10 molar ratio) for 20 min at room temperature and the fluorescence spectra were acquired as described previously [3], [8], [10]. As a control, the fluorescence spectra of individual proteins were also determined. For determination of interaction of Nk-PLA_2_ with PC in presence of 0.5 mM EDTA (to prevent the PC hydrolysis), our previously described procedure was followed [3]. The above experiments were repeated three times to assure the reproducibility.

### Platelet aggregation inhibition assay

For preparation of platelet rich plasma, citrated blood (1∶9, v/v) was centrifuged at 200× g for 20 min. The *in vitro* platelet aggregation inhibition property was investigated by pre-incubating different concentrations (0.5 to 2.0 µg) of purified PLA_2_ with thrombin (1 NIH U/ml) for 30 min at 37°C followed by induction of platelet aggregation. A control reaction was also set up where instead of purified Nk-PLA_2_, thrombin (1 NIH U/ml) was incubated with 1× PBS (20 mM K-phosphate, 150 mM NaCl, pH 7.4) at 37°C. The platelet aggregation was monitored in a Chrono-log dual channel aggregometer for 10 min. The experiment was repeated three times to assure the reproducibility.

### Effect of histidine modifier, heparin and commercial antivenom on enzymatic and anticoagulant activities

The PLA_2_ inhibitor (histidine modifier) p-bromophenacyl bromide (5 mM), heparin (1 IU) or graded amounts of monovalent antivenom (against *N. kaouthia* venom) were incubated with a fixed concentration of PLA_2_ (1.0 µg/ml) at 37°C for 30 min. The catalytic, anticoagulant, and thrombin/FXa inhibitory activity of PLA_2_ was then assayed as stated above. The activity of PLA_2_ in presence of its inhibitor/antivenom was thus compared with control (without inhibitor/antivenom).

### Statistical analysis

Results were presented as mean ± standard deviation (S.D.) of three experiments. Statistical analysis of the data was performed using a Student's t test in Sigma Plot 11 for Windows (version 7.0). Values of p≤0.05 were considered significant.

## Results

### Isolation and purification of two PLA_2_ isoenzymes from *N. kaouthia* venom

**Figure 1:**
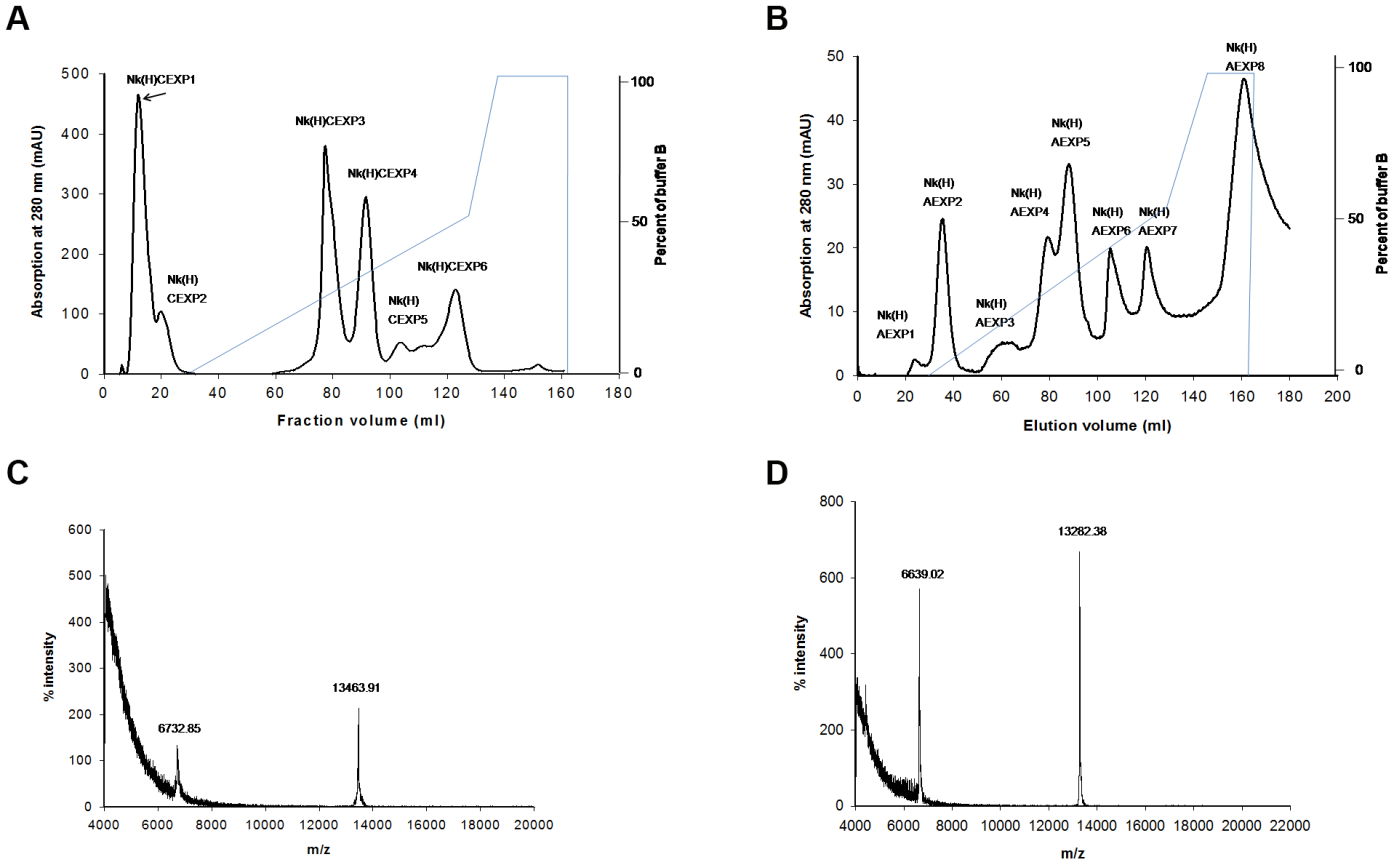
Purification and determination of molecular masses of PLA_2_ isoenzymes isolated from *N. kaouthia* venom. (**a**) Fractionation of crude *N. kaouthia* venom (25 mg dry weight) done on a HiPrep CM FF16/10 FPLC cation-exchange column. The fractionation conditions are described in the text. The peak showing major PLA_2_ and anticoagulant activity is marked with an arrow. (**b**) Chromatogram resulting from anion-exchange fractionation of cation-exchange unbound peak [Nk(H)CEXP1] by using a Hiprep DEAE FF16/10 FPLC column. (**c**) and (**d**) MALDI-TOF mass spectrum of Nk-PLA_2_α [peak Nk(H)AEXP4], and Nk-PLA_2_β [peak Nk(H)AEXP5], respectively.

Fractionation of crude *N. kaouthia* venom through Hiprep CM FF 16/10 column separated the proteins into 6 peaks, Nk(H)CEXP1 to Nk(H)CEXP6 (Fig. 1a).The peak 1 [Nk(H)CEXP1] eluted with the equilibration buffer showed a significant PLA_2_ as well as anticoagulant activity. By re-fractionation of this peak through Hiprep DEAE FF16/10 column, the proteins were separated into 8 fractions (Fig. 1b). The peak Nk(H)AEXP4 and Nk(H)AEXP5 demonstrated significant PLA_2_ as well as anticoagulant activity. The proteins of these peaks were found to be homogenous through MALDI-TOF-MS analysis with molecular mass of 13463.91 Da and 13282.38 Da, respectively (Figs. 1c–d). A summary of purification of these purified anticoagulant PLA_2_s, named Nk-PLA_2_α and Nk-PLA_2_β is shown in Table 1.

**Table 1:** A summary of the purification of PLA_2_ isoenzymes from the venom of *N. kaouthia*.

Fraction	Total protein (mg)	Yield of protein (%)	PLA_2_ activity		Purification
			Total activity	Specific activity	(fold)
			(Units)^a^	(Units/mg)	
Crude venom	16.1	100	24.2×10^4^	1.5×10^4^	1.0
Nk(H)CEXP1	1.5	9.3	2.6×10^4^	1.7×10^4^	1.1
Nk(H)AEXP4	0.1	0.6	0.4×10^4^	4.1×10^4^	2.7
(NK-PLA_2_α)					
Nk(H)AEXP5	0.2	1.2	1.1×10^4^	5.4×10^4^	3.6
(NK-PLA_2_β)					

The PLA_2_ activity was assayed by egg-yolk phospholipids hydrolysis method using 0.1 M Tris-HCl, pH 8.0 buffer. Data are from a typical experiment.

a One unit of PLA_2_ activity is defined as decrease of 0.01 absorbance at 740 nm per 10 min at 25°C.

### LC-MS/MS identification of proteins

By LC-MS/MS analysis, both the PLA_2_s under study showed highest similarity (score 463) with PLA_2_ (EC 3.1.1.4) III isolated from monocled cobra venom (accession no. 2144440) with 23.5% sequence coverage. Furthermore, the PLA_2_s under study also demonstrated similarity (18-15% sequence coverage) with PLA_2_s isolated from *Naja naja* (accession no. 395192), anticoagulant PLA_2_ from *N. n. sagittifera* (accession no. 66361231), and an acidic PLA_2_ natratoxin from *N. atra* (accession no. 209573225) venom samples. Besides, both these PLA_2_s were found to possess identical sites for trypsin cleavage and therefore, they demonstrated similar trypic fragmentation pattern. The NCBI BLASTP search of one of the tryptic peptides GGSGTPVDDLDR (Mr. 1188.5 Da) showed putative conserved domains of PLA_2_-like superfamily. Another tryptic peptide sequence NMIQCTVPSR (Mr. 1148.5 Da) demonstrated 100% sequence similarity with an acidic PLA_2_ enzymes natratoxin (accession no. A4S04) isolated from the Chinese cobra *N. atra* venom. Furthermore, another tryptic peptide sequence viz. NLYQFK (Mr. 812.4 Da) was found to contain the N-terminal sequence of both these PLA_2_ isoezymes purified from *N. kaouthia* venom.

### Substrate specificity, anticoagulant activity and inhibition of platelet aggregation

Both NK-PLA_2_s showed preferential hydrolysis of PC over PS or PE (data not shown), which is similar to the phospholipid hydrolysis pattern of PLA_2_s, which we previously isolated from the venom of *N. kaouthia* [5]–[7]. The specific activity of Nk-PLA_2_α and Nk-PLA_2_β towards PC was determined as 2.3×10^5^ and 4.5×10^5^ units/mg, respectively. Both enzymes displayed optimum activity at the pH range of 8.0–9.0 and at 37–45°C (data not shown). Like other snake venom PLA_2_ enzymes [6]–[8], heating Nk-PLA_2_α or Nk-PLA_2_β at 75°C for 30 min did not result in any significant decrease in catalytic activity of heated enzyme as compared to control (unheated) enzyme (data not shown).

**Figure 2:**
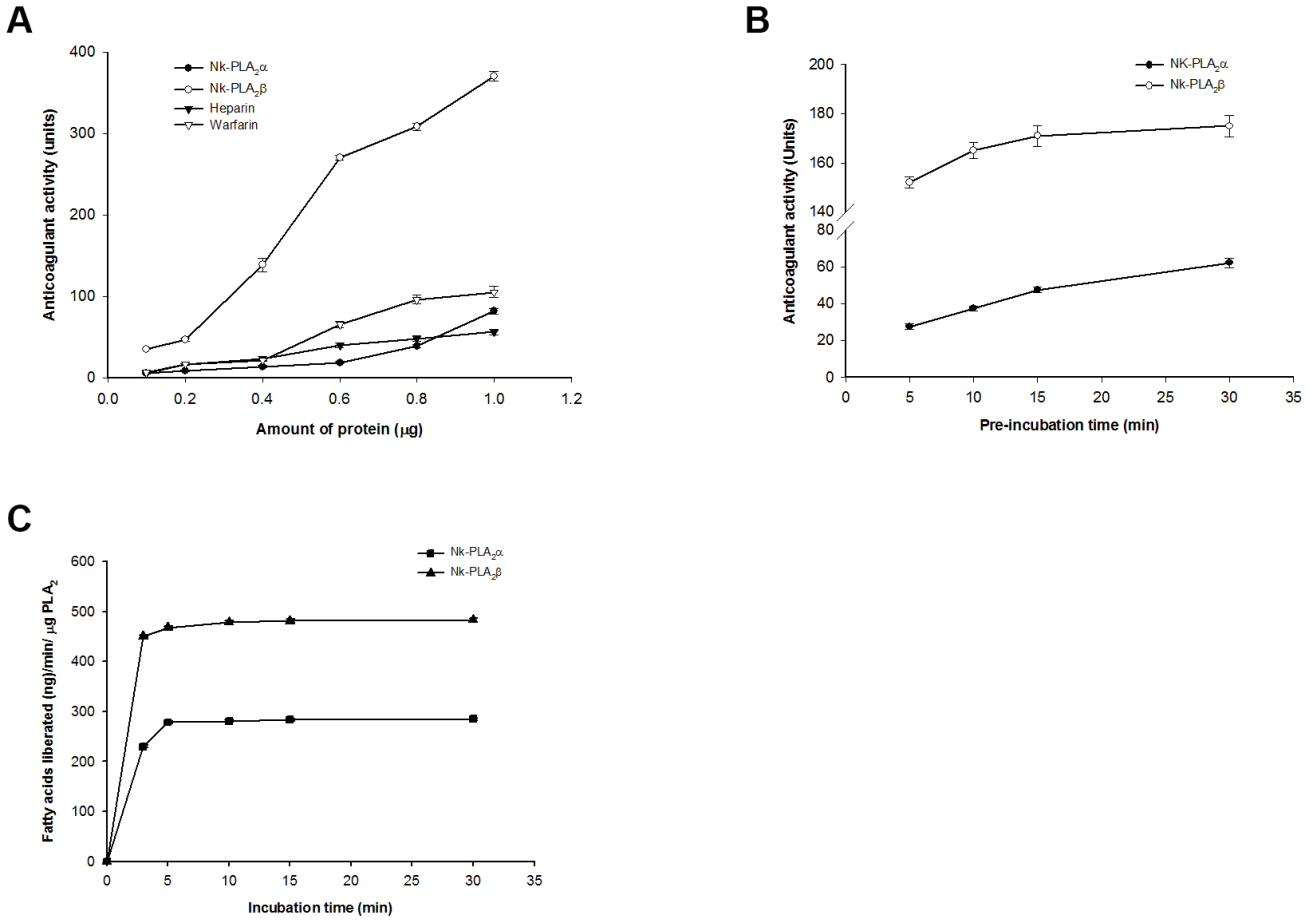
Comparison of anticoagulant activity and plasma phospholipids hydrolytic activity of Nk-PLA_2_α and Nk-PLA_2_β. (**a**) A comparison of dose-dependent anticoagulant activity (Ca-clotting time of citrated PPP) of Nk-PLA_2_α, Nk-PLA_2_β, heparin and warfarin. Unit is defined as 1s increase in clotting of PPP in presence anticoagulants compared to the clotting time of control PPP. (**b**) Effect of PPP/PLA_2_ pre-incubation time on anticoagulant activity. (**c**) Effect of PPP/PLA_2_ pre-incubation on release of free fatty acids from plasma phospholipids. Values are mean ± SD of triplicate determination.

A comparison of anticoagulant activity of the purified PLA_2_s with commercial anticoagulants heparin and warfarin is shown in Fig. 2a. Both these PLA_2_s increased the Ca-clotting time of PPP in a dose-dependent manner, however to a significantly different extent (Fig. 2a). The anticoagulant activity of Nk-PLA_2_β was found to be significantly higher (p<0.05) compared to Nk-PLA_2_α, heparin or warfarin under identical experimental conditions. It is worthy of mention that heparin shows anticoagulant effect by binding with antithrombin-III (AT-III) present in PPP, which in turn inhibits thrombin and activated factor X [16]. Pre-incubation of Nk-PLA_2_α (0.2 µg) and Nk-PLA_2_β (0.2 µg) at a ratio of 1∶1 (w∶w) for 20 min at room temperature prior to the addition of PPP did not allow the plasma to clot within 10 min. Conversely, the anticoagulant activity of individual Nk-PLA_2_α or Nk-PLA_2_β under identical experimental conditions was found to be much lower (Fig. 2a).

**Figure 3:**
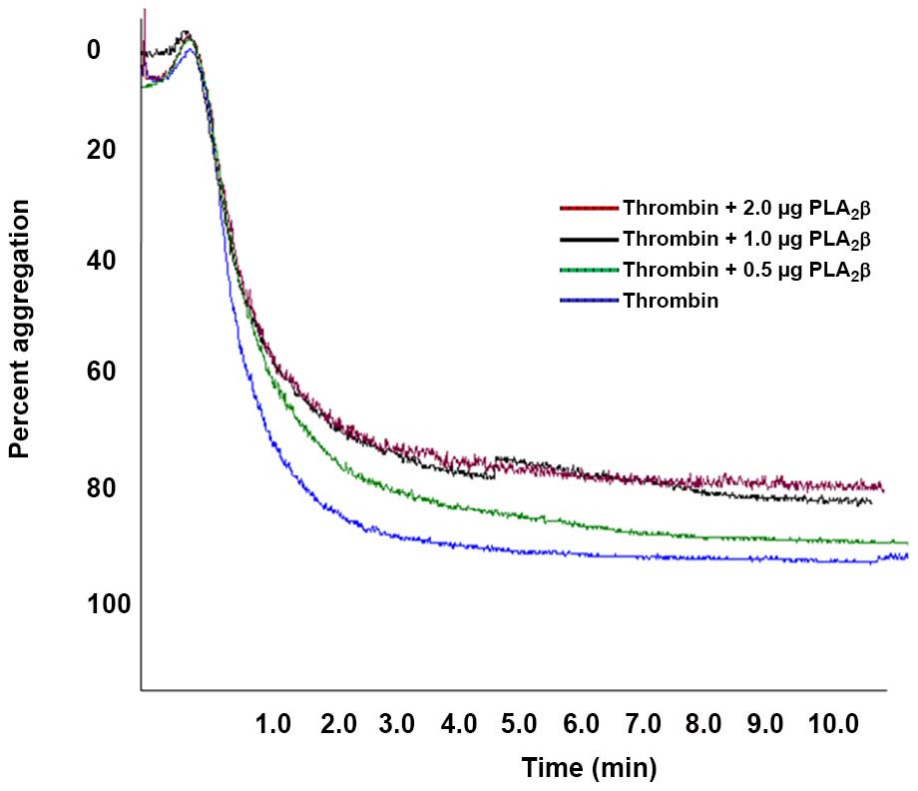
Inhibition of thrombin-induced platelet aggregation by different doses of Nk-PLA_2_β. The platelet aggregation was induced by with thrombin (1.0 NIH U/ml) pre-incubated with Nk-PLA_2_β (0.5–2.0 µg/ml) or 1× PBS (control) for 30 min at 37°C. The platelet aggregation was monitored in a Chrono-log dual channel aggregometer for 10 min. The data represent a typical experiment; however, the experiment was repeated three times to assure the reproducibility.

Increasing of the pre-incubation of PPP with Nk-PLA_2_α or Nk-PLA_2_β for 0–5 min before addition of CaCl_2_ enhanced the anticoagulant potency of both the enzymes (Fig. 2b). However, increasing of the pre-incubation of Nk-PLA_2_α or Nk-PLA_2_β with PPP from 5 to 30 min did not reveal any significant difference (*p*>0.05) in the release of fatty acids from plasma phospholipids (Fig. 2c). This result indicates that only a minor plasma phospholipids hydrolysis done by Nk-PLA_2_ isoenzymes is sufficient to exert their anticoagulant effect [17]. The Nk-PLA_2_α did not show inhibition of thrombin-induced platelet aggregation at a dose of 2.0 µg (data not shown). Conversely, Nk-PLA_2_β demonstrated dose-dependent partial inhibition of thrombin-induced platelet aggregation and saturation was observed at 2.0 µg PLA_2_ (Fig. 3).

### Inhibition of thrombin and FXa

**Figure 4:**
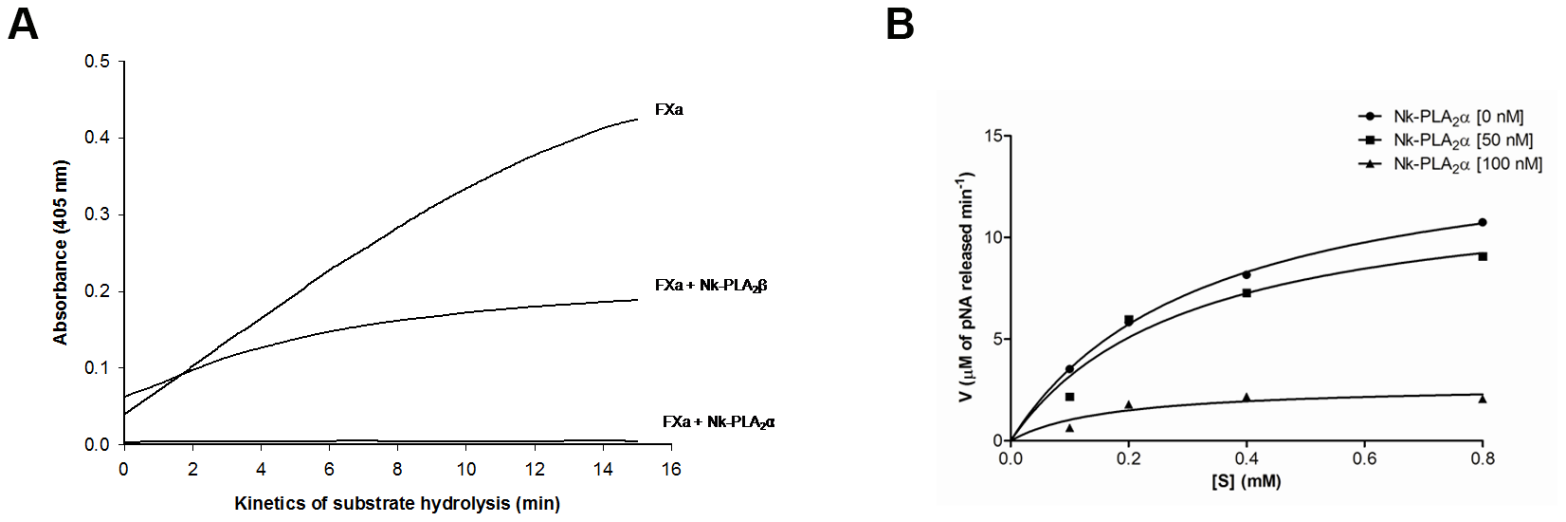
Effects of Nk-PLA_2_ isoenzymes on inhibition of amidolytic activity of FXa. (**a**)The Nk-PLA_2_α (1.0 µg) or Nk-PLA_2_β (1.0 µg) was pre-incubated with FXa (0.15 µM) against its chromogenic substrate F3301 (0.2 mM) for 60 min at 37°C, pH 7.4 before the amidolytic activity assay. A control (FXa treated with buffer) was also run in parallel. The values are mean of triplicate determinations. (**b**) Michaelis-Menten plot to determine the inhibitory constant (K_i_) of Nk-PLA_2_α (50 nM and 100 nM) on amidolytic activity of FXa (0.15 µM) at 37°C, pH 7.4.

**Figure 5:**
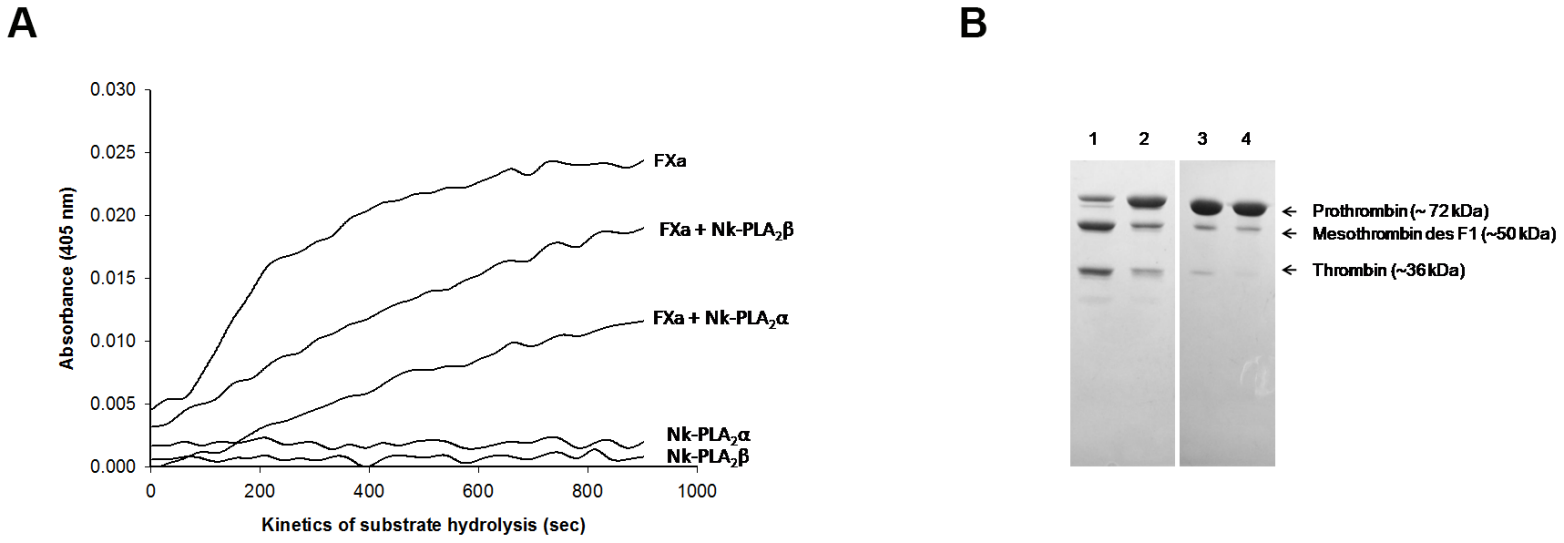
Inhibition of prothrombin activation by FXa pre-treated with NkPLA_2_ isoenzymes. (**a**) The FXa (20 nM) was pre-incubated with Nk-PLA_2_α (1.0 µg), or Nk-PLA_2_β (1.0 µg), or buffer (control) for 60 min at 37°C, pH 7.4. The prothrombin (1.4 µM) activation by FXa (treated or control) was determined by formation of thrombin by using its chromogenic substrate T1637 (0.2 mM). (**b**) SDS-PAGE (15%) analysis (reduced conditions) of affect of Nk-PLA_2_α and Nk-PLA_2_β on inhibition of prothrombin activation by FXa. Before addition of prothrombin (10.0 µg), FXa (20 nM) was pre-incubated with Nk-PLA_2_α or Nk-PLA_2_β for 60 min at 37°C, pH 7.4. Lane 1, prothrombin treated with FXa for 60 min at 37°C (control); lane 2, prothrombin treated with FXa (pre-incubated with 2.0 µg of Nk-PLA_2_β) for 60 min at 37°C; lanes 3 and 4, prothrombin treated with FXa (pre-incubated with 1 and 2 µg of Nk-PLA_2_α, respectively) for 60 min at 37°C. The experiment was repeated three times to assure the reproducibility.

None of the Nk-PLA_2_s showed amidolytic activity against the chromogenic substrate for FXa or thrombin. The Nk-PLA_2_α at a dose of 1.0 µg completely inhibited the amidolytic activity of FXa (0.15 µM); conversely, the Nk-PLA_2_β under identical experimental conditions displayed partial inhibition to FXa (Fig. 4a). The Michaelis-Menten plot showed (Fig. 4b) that Nk-PLA_2_α decreased in a dose-dependent manner, the *Km* as well as the *Vmax* values of factor Xa towards its chromogenic substrate (Table 2). The turnover number (*Kcat* value) was also found to decrease with an increase in inhibitor (Nk-PLA_2_α) concentration (Table 2). The *Ki* value for the inhibition of amidolytic activity of factor Xa by Nk-PLA_2_α was calculated at 42.1 nM. Furthermore, Nk-PLA_2_α was found to display a greater strength than Nk-PLA_2_β in inhibiting the prothrombin activation property of FXa (Fig. 5a). The SDS-PAGE analysis (under reduced conditions) of prothrombin degradation products by FXa in presence or absence of Nk-PLA_2_α or Nk-PLA_2_β also supports the above observation (Fig. 5b).

**Table 2:** Kinetics of inhibition of FXa (0.15 µM) and thrombin (0.03 NIH U/ml) by Nk-PLA_2_α and Nk-PLA_2_β at 37°C, pH 7.4, respectively.

Parameters	Factor Xa inhibition			Thrombin inhibition		
	Nk-PLA_2_α (nM)			Nk-PLA_2_β (nM)		
	0	50	100	0	50	100
*Vmax* (µmol pNA min^−1^)	15.05	12.72	2.76	1.68	0.378	0.054
*Km* (mM)	0.33	0.30	0.17	1.39	0.56	0.24
*Kcat* (min^−1^)	693	586	127	10	2.2	0.32

The kinetic parameters (*Km*, *Vmax*, and *Kcat*) values were determined from Michaelis-Menten plot as described in the text. The chromogenic substrates (0.2 mM) used for the amidolytic activity assay of thrombin and FXa were T1637 and F3301, respectively. The values are mean of triplicate determinations and SD was found within 10% of the mean value.

**Figure 6:**
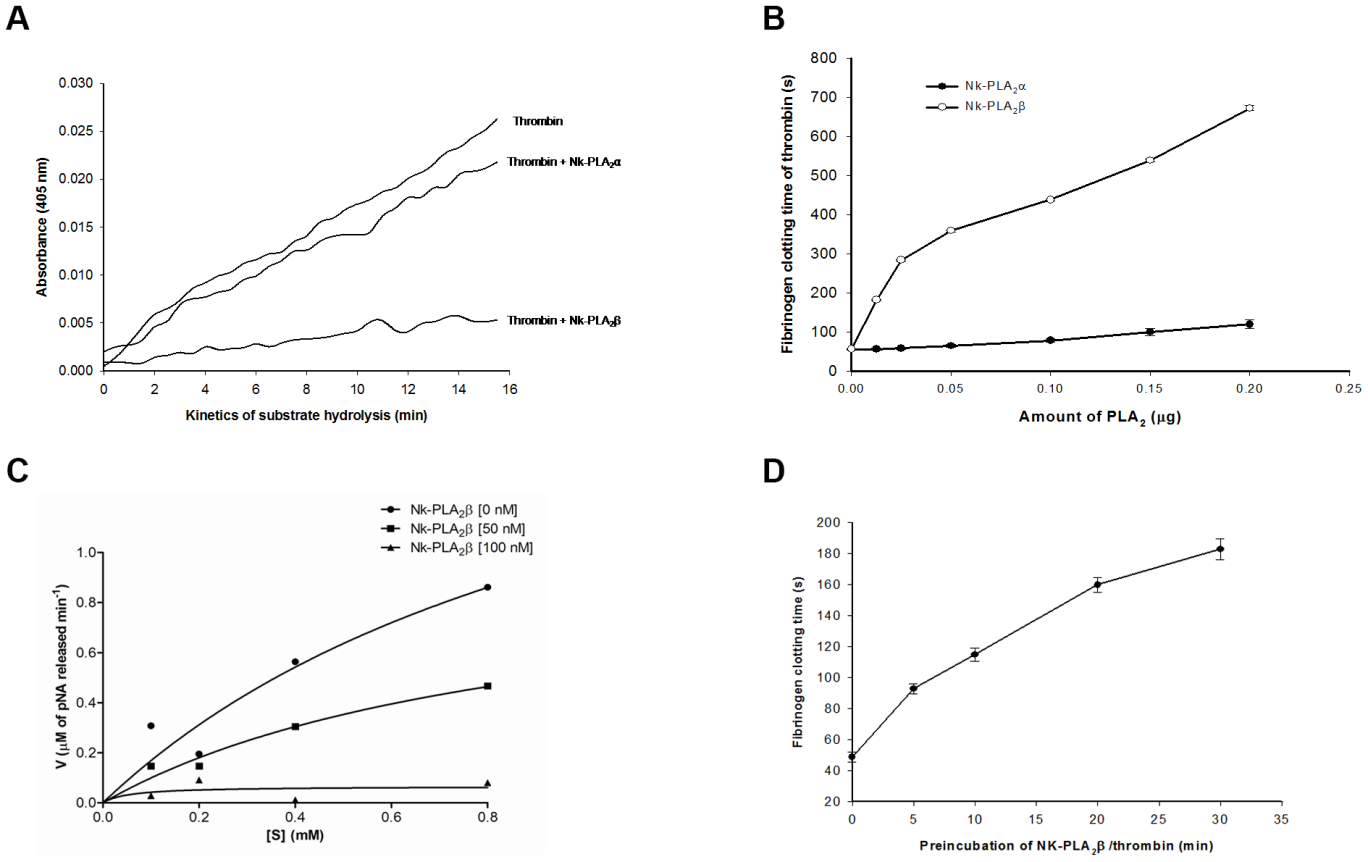
Inhibitory effect of Nk-PLA_2_α and Nk-PLA_2_β on amidolytic activity and fibrinogen clotting property of thrombin. (**a**) The thrombin (0.03 NIH U/ml) was pre-incubated with Nk-PLA_2_α (1.0 µg), or Nk-PLA_2_β (1.0 µg), or buffer (control) for 30 min at 37°C, pH 7.4 before addition of its chromogenic substrate T1637 (0.2 mM). The values are mean of triplicate determination. (**b**) Inhibition of fibrinogen clotting activity of thrombin through different doses (0–0.2 µg) of Nk-PLA_2_α or Nk-PLA_2_β. Before the addition of fibrinogen (final concentration ∼7.5 µM in 20 mM K-phosphate buffer, pH 7.4), thrombin (0.03 NIH U/ml) was pre-incubated with different doses of PLA_2_ (dissolved in 20 mM K-phosphate buffer, pH 7.4) for 30 min at 37°C. Values are mean ± SD of triplicate determination. (**c**) Michaelis-Menten plot to show the Inhibition of amidolytic activity of thrombin (0.03 NIH U/ml) through two different concentrations (50 nM and 100 nM) of Nk-PLA_2_β. Before the addition of substrate T1637 (0.2 mM), thrombin was pre-incubated with two different doses of the above PLA_2_ in 20 mM Tris-HCl, pH 7.4 for 30 min at 37°C (**d**) The effect of pre-incubation of Nk-PLA_2_β/thrombin on inhibition of fibrinogen clotting activity of thrombin. A fixed amount (0.05 µg) of Nk-PLA_2_β was pre-incubated with thrombin (0.03 NIH U/ml) for 0–30 min at 37°C and then assayed for fibrinogen clotting activity. The values are mean ± SD of triplicate determinations.

The Nk-PLA_2_ isoenzymes differentially inhibited the amidolytic activity (Fig. 6a) and fibrinogen clotting activity of thrombin (Fig. 6b). The Michaelis-Menten plot for the inhibition of amidolytic activity of thrombin at two different concentrations of Nk-PLA_2_β is shown in Fig. 6c. The Nk-PLA_2_β dose-dependently decreased the *Km*, *Vmax, and Kcat* values of thrombin towards its chromogenic substrate (Table 2). The *K_i_* value for thrombin inhibition displayed by Nk-PLA_2_β was determined at 9.4 nM. It was observed that under identical experimental conditions, the inhibition of fibrinogen clotting activity of thrombin done by Nk-PLA_2_β was more pronounced (p<0.01) compared to the same activity displayed by Nk-PLA_2_α (Fig. 6b). Increasing of the time (5–30 min) during which Nk-PLA_2_β was pre-incubated with thrombin (prior to addition of fibrinogen) linearly decreased the fibrinogen clotting activity (or increased the fibrinogen clotting time) of thrombin (Fig. 6d).

### Spectrofluorometric analysis of binding of PLA_2_s with thrombin, FXa or PC

**Figure 7:**
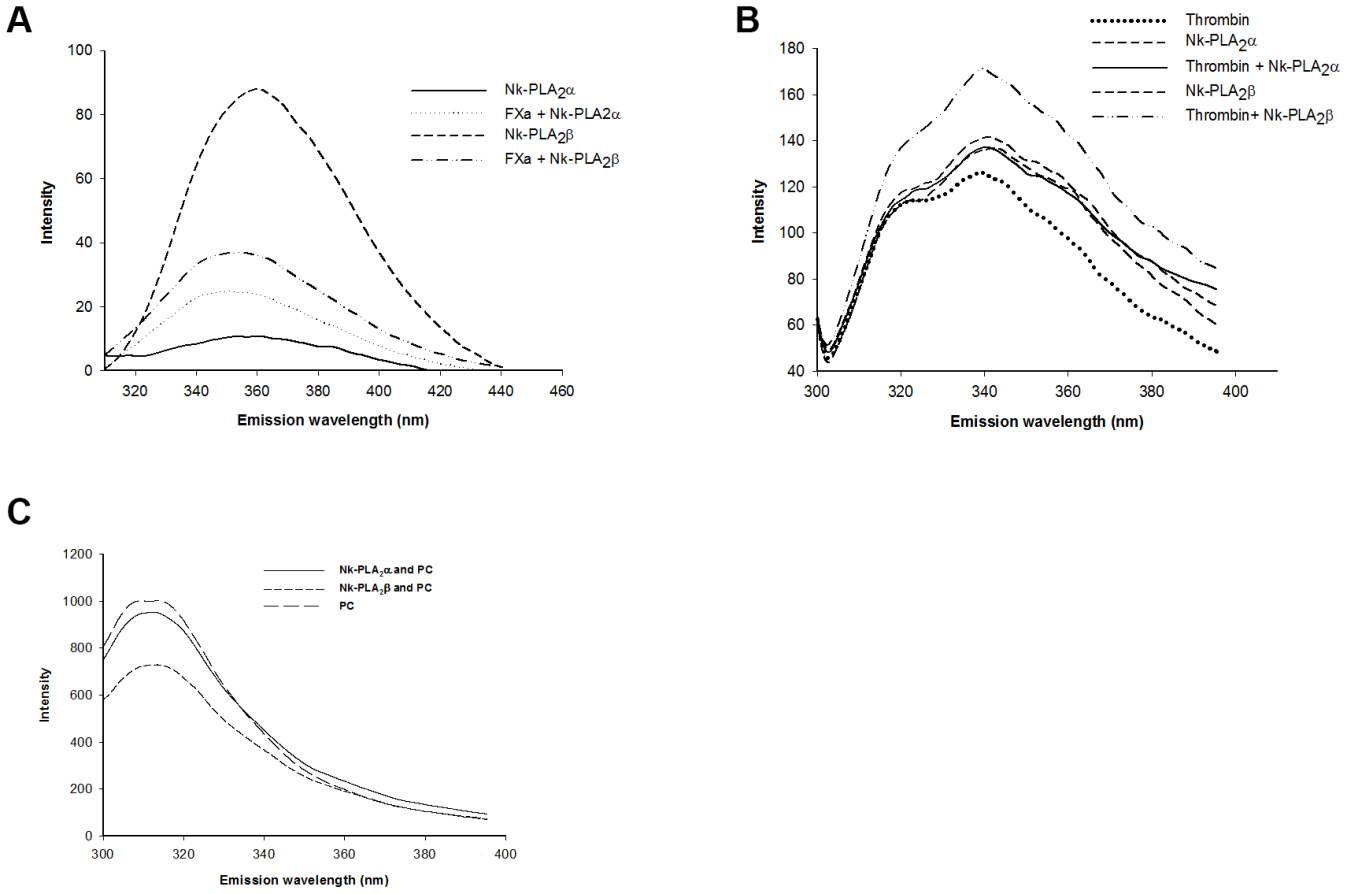
Spectrofluorometric assay of the interaction of Nk-PLA_2_ isoenzymes with FXa, thrombin and PC. (**a**) FXa incubated with Nk-PLA_2_α or Nk-PLA_2_β (at 1∶10 ratio), (**b**) interaction of thrombin with Nk-PLA_2_α or Nk-PLA_2_β (at 1∶10 ratio), (**c**) PC with Nk-PLA_2_α/Nk-PLA_2_β (at 10∶1 ratio) in presence of 0.5 mM EDTA (to prevent the phospholipids hydrolysis). The data shown above represent a typical experiment; however, the experiments were repeated three times to assure the reproducibility.

The interaction of FXa with Nk-PLA_2_α (at 1∶10 ratio) resulted in an increase in the emission of fluorescence signal (measured using excitation at 280 nm) of Nk-PLA_2_α–FXa complex (Fig. 7a). However, the interaction of Nk-PLA_2_β with FXa decreased the fluorescence signal of Nk-PLA_2_β-FXa complex compared to the fluorescence signal produced by Nk-PLA_2_β (Fig. 7a). Moreover, the fluorescence signal at ∼345 nm produced by Nk-PLA_2_β was found to be higher than that of Nk-PLA_2_α suggesting presence of more tryptophan residue in the former PLA_2_ enzyme. The interaction of Nk-PLA_2_α or Nk-PLA_2_β with thrombin showed that the intrinsic fluorescence of Nk-PLA_2_β was significantly increased in the presence of thrombin (Fig. 7b); conversely, interaction of Nk-PLA_2_α with thrombin influenced the fluorescence signal only to a marginal extent (Fig. 7b).

The effect of interaction between Nk-PLA_2_α/Nk-PLA_2_β and PC is shown in Fig. 7c. The interaction between Nk-PLA_2_α and PC in the presence of EDTA (to prevent the PC hydrolysis) resulted in a small decrease in the emission intensity with respect to the emission strength of PC alone. However, interaction effect of Nk-PLA_2_β with PC was found to be more pronounced because a large decrease in emission intensity was observed (Fig. 7c).

### Effect of histidine modifier (PLA_2_ inhibitor) and antivenom on catalytic and anticoagulant activity

Like other snake venom PLA_2_ enzymes, the catalytic as well as anticoagulant activity of Nk-PLA_2_α and Nk-PLA_2_β was drastically and simultaneously reduced by pBPB, a modifier of histidine (Table 3). The monovalent antivenom was found to inhibit the catalytic and anticoagulant activity of Nk-PLA_2_α and Nk-PLA_2_β to a significantly (p<0.01) different extent. However, at 1∶10 antigen (PLA_2_): antivenom (protein∶protein) ratio the catalytic activity of both the enzymes were inhibited to 62 to 65% of their original activity whereas their anticoagulant activity was not affected (Table 3). At an antigen (PLA_2_): antivenom ratio of 1∶200, the monovalent antivenom also failed to neutralize the thrombin inhibitory activity of these PLA_2_ enzymes (Table 3). Interestingly, heparin showed dissimilar effect in neutralizing the catalytic property of Nk-PLA_2_α and Nk-PLA_2_β isoenzymes; nevertheless, FXa or thrombin inhibitory properties of these PLA_2_ isoenzymes in presence of heparin remained unaffected (Table 3).

**Table 3:** Effect of pBPB and monovalent antivenom on catalytic, anticoagulant and thrombin inhibitory activity of Nk-PLA_2_α and Nk-PLA_2_β.

Inhibitor/antivenom	Percent inhibition					
	Catalytic		Anticoagulant		FXa inhibition	Thrombin inhibition
	Nk-PLA_2_α	Nk-PLA_2_β	Nk-PLA_2_α	Nk-PLA_2_β	Nk-PLA_2_α	Nk-PLA_2_β
pBPB (5 mM)	68.7±2.1	81.6±3.2^a^	42±2.2	51.8±3.4^b^	ND	ND
Heparin (1 IU)	23.7±2.1	6.8±1.0^a^	16.1±1.8	0^a^	0	0
Monovalent antivenom						
1∶10	65.0±3.3	62.0±4.2	0	0	0	0
1∶100	98±1.0	96±2.1	0	0	0	0
1∶200	100	100	21.3±1.5	19.8±2.1	18.7±2.0	13.5±1.7^b^

The activity of enzymes in absence of inhibitor/antivenom was considered as 100% activity and the other values were compared with that. The values are mean ± S. D. of triplicate determinations. ND: not determined; pBPB: p-bromophenacyl bromide. Significance of difference with respect to Nk-PLA_2_α;

a p<0.01,

b P<0.05.

## Discussion

The LC-MS/MS analysis of both these PLA_2_ isoenzymes has suggested their identity with PLA_2_ enzymes isolated from cobra venom. The molecular mass of Nk-PLA_2_α (13,463.91 Da) and Nk-PLA_2_β (13,282.38 Da) is very close to the molecular mass of previously reported PLA_2_ isoenzymes NK-PLA_2_-I (13.6 KDa) and Nk-PLA_2_-A (13,619.36 Da), and Nk-PLA_2_-II (13,346.19 Da) and Nk-PLA_2_-B (13,303.05 Da), isolated from venom samples of *N. kaouthia* of different geographical locations [5]–[7]. Moreover, the active sites of Nk-PLA_2_α and Nk-PLA_2_β, like classical PLA_2_ enzymes from snake venom, also contain histidine which is considered as the most crucial residue for phospholipids hydrolysis [3], [5]–[8], [10], [18]. These combined data suggest that Nk-PLA_2_α and Nk-PLA_2_β are PLA_2_ isoenzymes purified from *N. kaouthia* venom of eastern India origin.

The PLA_2_ enzymes on the basis of their potencies to prolong the re-calcification time of PPP have been classified into weak or strong anticoagulant enzymes [2], [19]. The anticoagulant strength of Nk-PLA_2_α or Nk-PLA_2_β categorized them to strong anticoagulant PLA_2_ enzymes [2]–[3], [8], [10]. The significantly higher anticoagulant activity of Nk-PLA_2_β than that of Nk-PLA_2_α suggests that these PLA_2_ isoenzymes have evolved to exert pharmacological effects by different mechanisms in snakebite victims. Recently Saul et al [20] have also reported the presence of two PLA_2_ isoforms in venom of *Vipera ammodytes ammodytes*, which differ in sequence by only two amino acid residues (Phe^124^ is replaced by Ile^124^ and Lys^128^ is replaced by Glu^128^). These natural PLA_2_ isoforms exhibit significant differences in toxicity and anticoagulant potencies [20]. Comparison of primary structures of both the Nk-PLA_2_s, which is our next goal of study, may shed light on their structural motifs correlated to the differences in their biological activity.

Snake venom PLA_2_s enzymes most likely prolong the blood coagulation through hydrolysis of pro-coagulant phospholipids of plasma rendering them unavailable for initiating the coagulation process [2]–[3], [8], [10]. The role of catalytic activity in anticoagulant mechanism is evident from the fact that Nk-PLA_2_β compared to Nk-PLA_2_α demonstrating superior PC binding as well as PC hydrolysis property also shows higher anticoagulant potency [2]–[3], [8]. Furthermore, inhibition study with heparin and pBPB also supports the role of catalytic activity in anticoagulant property of NK-PLA_2_ isoenzymes. It has been mentioned that very low but specific plasma phospholipids hydrolysis is the characteristics feature of strongly anticoagulant PLA_2_s whereas non specific, non-anticoagulant PLA_2_ enzymes hydrolyze the plasma phospholipids indiscriminately and therefore, they fail to retard the blood coagulation [2]–[3], [10], [17]. Furthermore, these results are in accordance with our previous observations showing strong anticoagulant PLA_2_ enzymes affect blood coagulation by mechanism that is independent of phospholipids hydrolysis [3], [8]. Differential inhibition of catalytic activity and anticoagulant property (pharmacological activity) of Nk-PLA_2_α and Nk-PLA_2_β by monovalent antivenom, pBPB (histidine modifier, PLA_2_ inhibitor) and heparin suggests that there are separate or perhaps overlapping sites in these PLA_2_ molecules that contribute to these activities [2]–[3], [21]. Studies have indicated that heparin acts as a competitive inhibitor for human secretory class II PLA_2_ and closely related snake venom PLA_2_ enzymes [22].

The non-enzymatic mechanism of anticoagulant action of snake venom PLA_2_ has been executed by competing with blood clotting factors such as thrombin, FXa, Va, or prothrombinase complex in the lipid surface [2]–[3], [8], [11]–[12], [20]. The present study strongly indicates that Nk-PLA_2_α or Nk-PLA_2_β prolongs the blood coagulation by targeting different coagulation factors. The anticoagulant mechanism of Nk-PLA_2_β is partially dependent on FXa inhibition suggesting FXa is the major pharmacological target for Nk-PLA_2_α to disrupt the coagulation cascade through a non-catalytic, phospholipids independent mechanism. This significant difference in potency of FXa inhibition by two PLA_2_ isoenzymes purified from the same snake venom is in accordance with the report of Saul et al. [20]. It has been shown that a point mutation of Lys^128^ to Glu^128^ perturbs the interaction of PLA_2_ isoform with FXa results in a 10-fold decrease in the affinity of mutated enzyme for FXa [20]. Therefore, it may be anticipated that small surface residue changes in snake venom isoenzymes can give rise to very significant changes in their biological activity [15], [20], [23]. The kinetics of inhibition suggests that the Nk-PLA_2_α binds to the enzyme (FXa)-substrate complex (uncompetitive mode of inhibition) rather than competing with the substrate for binding at the active site of the enzyme. In contrast, strongly anticoagulant PLA_2_ enzyme CM-IV purified from *N. nigricollis* venom is a non-competitive inhibitor of FXa [24]. Furthermore, inhibition of FXa by Nk-PLA_2_α is more pronounced compared to CM-IV (*Ki* = 250 nM) [24]. Moreover, the mechanism of FXa inhibition by Nk-PLA_2_α or Nk-PLA_2_β and human group II secretory PLA_2_ (hsPLA_2_) seems to be different because hsPLA_2_ does not inhibit the amidolytic activity of FXa [25].

The Nk-PLA_2_β exerts potent anticoagulant activity via targeting thrombin without the requirement of any cofactor. Till date, only two snake venom PLA_2_ enzymes, one purified from venom of *N. haje* [11] and the other from the venom of *Daboia russelii russelii* [3], have been demonstrated to prolong PPP clotting via thrombin inhibition. The *Ki* value indicates that Nk-PLA_2_β is a more potent inhibitor of thrombin compared to the previously reported thrombin-inhibitor PLA_2_s isolated from snake venoms [3], [11]. Furthermore, Nk-PLA_2_β inhibited thrombin by uncompetitive mechanism whereas TI-Nh, a PLA_2_ purified from *N. haje* venom is a mixed-type inhibitor of thrombin [11]. Therefore, the mechanism and strength of thrombin inhibition by PLA_2_ enzymes purified from venoms of *N. haje* and *N. kaouthia* are different. The spectrofluorometric data also vouch the interaction of Nk-PLA_2_α and Nk-PLA_2_β with FXa and thrombin [3], [8]–[9]. However, the significantly different strength of binding of these PLA_2_ isoenzymes to thrombin or FXa reinforces the fact that these PLA_2_ isoenzymes have different pharmacological targets in order to exert their anticoagulant action [2]. Moreover, like RVAPLA_2_ isolated from *D. r. russelii* venom [3], the binding of Nk-PLA_2_β to thrombin is not a very rapid event. In a sharp contrast, a Kunitz-type protease inhibitor isolated from *D. r. russelii* venom binds to thrombin quickly [26].

The final stage of the blood coagulation cascade is catalyzed by thrombin, a 36 kDa trypsin-like serine protease, which is synthesized in the liver as its precursor prothrombin. Inactive prothrombin is proteolytically cleaved by the prothrombinase complex involving the activated factor X (FXa)/Va to form thrombin which is responsible for physiologic homeostasis and pathologic thrombosis [27]. Thrombin and FXa are, therefore, important pharmaceutical targets for the treatment and prevention of thrombotic disorder, a leading cause of cardiovascular death throughout the world [28]–[29]. Furthermore, platelet aggregation inhibitor aspirin prevents intravascular clot formation and it has therefore been used as prophylaxis to prevent myocardial infarction and stroke for more than 100 years now. However, several limitations of aspirin have been reported and the best dose of aspirin for the prevention of cardiovascular disease has not been well established [30]. The Nk-PLA_2_β by virtue of its thrombin inhibitory property partially prevented the thrombin-mediated aggregation of human platelets which may have therapeutic implications for averting unwanted clot formation.

Despite their widespread use, the anticoagulant drugs heparin and warfarin are associated with significant complications [28]–[29]. This underscores the search for new, safe and potent anticoagulant drugs that do not depend on vitamin K antagonism [29]. Notably, the cardiovascular therapeutic area has been undergoing unprecedented changes with the clinical introduction of new drugs targeting thrombin, factor Xa and inhibition of platelet aggregation. The direct thrombin inhibitors compared to indirect thrombin inhibitor such as heparin may offer a more specific anticoagulant effect [26]. Furthermore, our previous studies had vouched the non-toxic nature of *N. kaouthia* PLA_2_ isoenzymes [5], [7]. Taken together, it may be anticipated that future therapeutic application of Nk-PLA_2_α and Nk-PLA_2_β as combined platelet aggregation inhibitors and anticoagulant agents for the treatment and/or prevention of cardiovascular disorder is highly promising [13], [28].
